# Development and characterization of a fecal-induced peritonitis model of murine sepsis: results from a multi-laboratory study and iterative modification of experimental conditions

**DOI:** 10.1186/s40635-023-00533-3

**Published:** 2023-07-17

**Authors:** Neha Sharma, Damian Chwastek, Dhruva J. Dwivedi, Jared Schlechte, Ian-Ling Yu, Braedon McDonald, Jaskirat Arora, Erblin Cani, Mikaela Eng, Doreen Engelberts, Eva Kuhar, Sarah K. Medeiros, Stephane L. Bourque, Gediminas Cepinskas, Sean E. Gill, Forough Jahandideh, Kimberly F. Macala, Sareh Panahi, Cynthia Pape, David Sontag, Janet Sunohara-Neilson, Dean A. Fergusson, Alison E. Fox-Robichaud, Patricia C. Liaw, Manoj M. Lalu, Asher A. Mendelson, for the National Preclinical Sepsis Platform, Sepsis Canada

**Affiliations:** 1 Thrombosis and Atherosclerosis Research Institute, Hamilton, ON Canada; 2 Department of Medical Sciences, McMaster University, Hamilton, ON Canada; 3 Regenerative Medicine Program, Ottawa Hospital Research Institute, Ottawa, ON Canada; 4 Department of Medicine, McMaster University, Hamilton, ON Canada; 5 Snyder Institute for Chronic Diseases, Cumming School of Medicine, University of Calgary, Calgary, AB Canada; 6 Department of Critical Care Medicine, Cumming School of Medicine, University of Calgary, Calgary, AB Canada; 7 Department of Anesthesiology and Pain Medicine, University of Alberta, Edmonton, AB Canada; 8 Centre for Critical Illness Research, Lawson Health Research Institute, London, ON Canada; 9 Department of Medical Biophysics, Schulich School of Medicine and Dentistry, Western University, London, ON Canada; 10 Department of Physiology and Pharmacology, Schulich School of Medicine and Dentistry, Western University, London, ON Canada; 11 Department of Critical Care Medicine, Royal Alexandra Hospital, University of Alberta, Edmonton, AB Canada; 12 Department of Medicine, Section of Critical Care Medicine, Rady Faculty of Health Sciences, University of Manitoba, Health Sciences Centre Winnipeg, Rm GF-234, 820 Sherbrook St, Winnipeg, MB R3A 1R9 Canada; 13 Animal Care and Veterinary Services, Western University, London, ON Canada; 14 Faculty of Medicine, University of Ottawa, Ottawa, ON Canada; 15 Clinical Epidemiology Program, Blueprint Translational Group, Ottawa Hospital Research Institute, 501 Smyth Road, P.O. Box 201B, Ottawa, ON K1H 8L6 Canada; 16 Department of Anesthesiology and Pain Medicine, The Ottawa Hospital, Ottawa, ON Canada

**Keywords:** Sepsis, Preclinical sepsis, Animal models of sepsis, Fecal-induced peritonitis, National Preclinical Sepsis Platform, Multi-laboratory, Preclinical reproducibility

## Abstract

**Background:**

Preclinical sepsis models have been criticized for their inability to recapitulate human sepsis and suffer from methodological shortcomings that limit external validity and reproducibility. The National Preclinical Sepsis Platform (NPSP) is a consortium of basic science researchers, veterinarians, and stakeholders in Canada undertaking standardized multi-laboratory sepsis research to increase the efficacy and efficiency of bench-to-bedside translation. In this study, we aimed to develop and characterize a 72-h fecal-induced peritonitis (FIP) model of murine sepsis conducted in two independent laboratories. The experimental protocol was optimized by sequentially modifying dose of fecal slurry and timing of antibiotics in an iterative fashion, and then repeating the experimental series at site 1 and site 2.

**Results:**

Escalating doses of fecal slurry (0.5–2.5 mg/g) resulted in increased disease severity, as assessed by the modified Murine Sepsis Score (MSS). However, the MSS was poorly associated with progression to death during the experiments, and mice were found dead without elevated MSS scores. Administration of early antibiotics within 4 h of inoculation rescued the animals from sepsis compared with late administration of antibiotics after 12 h, as evidenced by 100% survival and reduced bacterial load in peritoneum and blood in the early antibiotic group. Site 1 and site 2 had statistically significant differences in mortality (60% vs 88%; *p* < 0.05) for the same dose of fecal slurry (0.75 mg/g) and marked differences in body temperature between groups.

**Conclusions:**

We demonstrate a systematic approach to optimizing a 72-h FIP model of murine sepsis for use in multi-laboratory studies. Alterations to experimental conditions, such as dose of fecal slurry and timing of antibiotics, have clear impact on outcomes. Differences in mortality between sites despite rigorous standardization warrants further investigations to better understand inter-laboratory variation and methodological design in preclinical studies.

**Supplementary Information:**

The online version contains supplementary material available at 10.1186/s40635-023-00533-3.

## Introduction

Multiple factors contribute to the lack of bench-to-bedside translational success for preclinical sepsis research, including the complex and heterogenous nature of sepsis and methodological challenges with preclinical experimentation [1–3]. Common criticisms of animal models of sepsis include the use of genetically identical, young, and predominantly male mice with no comorbidities, which do not adequately represent human sepsis [1, 4–6]. Animal studies of sepsis have also been criticized for not reflecting the clinical setting, such as the lack of routine pharmacological and supportive therapies (e.g., fluid resuscitation and antibiotics) and timing of interventions (e.g., novel therapeutics as a pre- or co-treatment with septic inoculation) [1, 7, 8]. A lack of standardization of preclinical sepsis models is also a concern, with numerous models being used interchangeably—endotoxin/lipopolysaccharide model, fecal pellet model, bacterial inoculum, cecal ligation and puncture model, and colon ascendant stent peritonitis model [2, 9, 10]. Even within the same model, many protocol variations exist between groups, and transparency of methodological reporting is poor [1, 2, 11]. The impact of these protocol variations on experimental outcomes is not well-described. Furthermore, these studies are often conducted in single centers which limits their generalizability and reproducibility [12–14]. Collectively, these factors contribute to the translational “valley of death”—the gap between bench research and clinical application [15]—whereby no experimentally derived sepsis therapies are currently approved for routine use in clinical practice [16].

To address these deficiencies and improve the quality of preclinical sepsis research, we established the National Preclinical Sepsis Platform (NPSP) as part of Sepsis Canada [1]. Sepsis Canada is a pan-Canadian research network funded by the Canadian Institutes of Health Research with a mandate to create infrastructure and study sepsis across all domains of inquiry, including biomedical research [1]. Within Sepsis Canada, the NPSP is a collaborative network of Canadian basic science researchers with a collective goal of conducting multi-laboratory preclinical studies on sepsis [1]. Recognizing the importance of developing a standardized approach in sepsis modeling to improve translational impact, we have incorporated the recommendations provided by the Wiggers–Bernard conference minimum quality threshold in preclinical sepsis studies (MQTiPSS) [2]. To assess generalizability of findings, we adopted a multi-laboratory preclinical approach, where laboratories use common shared models, protocols, and outcomes for evaluation. While multi-center studies are considered the gold standard in clinical research, their importance has only recently been recognized and incorporated in the preclinical setting [17].

Our aim was to develop and characterize a 72-h fecal-induced peritonitis (FIP) model of murine sepsis with a targeted mortality of 30–40%—consistent with mortality rates for septic shock in humans [18–20]. Unlike other murine models of sepsis, the FIP model minimizes surgical variability, is less operator-dependent, and is therefore easier to reproduce in a multi-laboratory setting [9, 10]. Moreover, most preclinical sepsis models are either acute or chronic, but do not capture the subacute phase of sepsis [21–25], which may have important pathophysiological relevance. We implemented a study design within a standardized multi-laboratory format that iteratively modifies experimental variables to determine how these changes impacted animal physiology and surrogate outcomes for mortality. Furthermore, we evaluated the degree of experimental variability between two sites conducting the same experimental protocol. These experiments will allow us to refine the logistics needed for coordinating future multi-laboratory preclinical trials.

## Methods

Mice received humane care in accordance with Canadian Council on Animal Care (CCAC) guidelines and all studies were approved by local Animal Research Ethics Board. This study is reported in accordance with the ARRIVE 2.0 guidelines (Additional file 2: Appendix 1).

### Animals

Male and female C57BL/6 mice (*Helicobacter hepaticus-free*; 8–10 weeks of age weighing approximately 20–30 g) were purchased from Charles River Laboratories (Sherbrooke, Quebec, Canada) and placed in standard housing in the Animal Care Facility at the Thrombosis and Atherosclerosis Research Institute (TaARI) at McMaster University (Hamilton, Ontario, Canada) and at the University of Ottawa (Ottawa, Ontario, Canada), herein denoted as site 1 and site 2, respectively. Mice were housed in a positive Helicobacter and Norovirus room in HEPA-filtered ventilated cages (Tecniplast Sealsafe Plus system) under 12-h dark/light cycles. Cages contained corncob bedding, nesting material, a structure (e.g., plastic igloos), autoclaved bottled water (provided via the Avidity Life Sciences Reverse Osmosis 8600 system), and food (Teklad Irradiated Global 18% Protein Rodent Diet 2918) ad libitum. Animals were housed with 1 to 4 mice per cage and acclimatized for at least 1 week prior to use in 72-h survival studies. At site 1, the ambient temperate range for all rodent areas was 20–22 °C and the humidity was set between 30 and 60%. At site 2, the ambient temperature range for all rodent areas was 21–24 °C, and the humidity was set between 30 and 60%.

### Experimental sepsis: fecal-induced peritonitis (FIP) model of sepsis

The fecal slurry was prepared from male Sprague Dawley rats, approximately 9 weeks of age, purchased from Charles River Laboratories (Sherbrooke, Quebec, Canada). Rat species were selected to maximize the slurry yield per animal, recognizing that our need for fecal inoculation with enteric microbes was not species-dependent. After rats were euthanized by excision of the heart, the cecal contents were collected. The cecal contents were homogenized in 50 mM phosphate buffer (6 mL/g) and a 30 mL syringe was used to break up the contents. The slurry was filtered using a 100 μM cell strainer to remove large particles and centrifuged at 3000 × g for 25 min at 4 °C. The pellet was resuspended in 5% dextrose solution (with 10% glycerol) to generate a final concentration of 100 mg/mL. Resuspended slurry from various conical tubes were combined in a beaker and stirred. Any remaining particles were broken up with a 30 mL syringe. The fecal slurry was prepared at site 1 in large batches to minimize variation of the contents between experiments and were aliquoted (1 mL per tube) and stored at − 80 °C.

The rat fecal slurry or control vehicle (5% dextrose solution with 10% glycerol) was injected into healthy male and female C57BL/6 mice, 9–13 weeks of age, and weighing approximately 20–30 g. Because this was a study designed for model development, we did not undertake blinding or randomization for group allocation. The required aliquots of fecal slurry were thawed and warmed to room temperature. Mice received an intraperitoneal (IP) injection of fecal slurry under isoflurane anesthesia into the right or left lower abdominal quadrant using a syringe and 25-gauge needle at doses of 0.5 mg/g, 0.625 mg/g, 0.75 mg/g, 1 mg/g, 1.5 mg/g, or 2.5 mg/g according to body weight and experimental protocol. Control mice received an IP injection of the control vehicle. Following injection, mice from the same treatment group were kept together and returned to their cages with bedding, enrichment, food, and water and allowed to recover. External heat was provided for all mice through heating blankets placed below half of each cage to allow mice to regulate their own body temperature [26].

### Supportive treatment

Fluids, antibiotics, and analgesia were administered to all mice, consistent with standard practice for the treatment of human sepsis and current MQTiPSS recommendations [1, 2]. Modification to the timing, dose, and class of antibiotic varied as the experimental protocol was being developed (Table 1). Fluids were administered as subcutaneous Ringer’s lactate; analgesia was given as subcutaneous buprenorphine; antibiotics were prescribed as either IP piperacillin–tazobactam or imipenem in 100 μL of Ringer’s lactate (Table 1). In general, fluids and analgesia were given at the same time as antibiotics to minimize handling and stress.

**Table 1:** An outline of our experimental design

Series#	Site	Fecal slurry batch	Fecal slurry dose: (mg/g)	Analgesia	Fluids	Antibiotics
1	1	2021	0.5, 0.75, 1, 1.5, 2.5	Buprenorphine (0.01 mg/kg) (4 h, 16 h, q8h onwards)	Ringer’s (200 µL, 4 h) Ringer’s (100 µL. 16 h, q8h onwards)	Piptazo (25 mg/kg) (8 h, q8h onwards)
2	1	2021	0.75	Buprenorphine (0.05 mg/kg) (4 h, 12 h, q12h onwards)	Ringer’s (20 mL/kg) (4 h, 12 h) Ringers (15 mL/kg) (24 h, q12h onwards)	Imipenem (25 mg/kg) (4 h, 12 h, q12h onwards)
3	1	2021	0.75	Buprenorphine (0.05 mg/kg) (4 h, 12 h, q12h onwards)	Ringer’s (15 mL/kg) (12 h, q12h onwards}	Imipenem (25 mg/kg) (12 h, q12h onwards)
3	2	2021	0.5, 0.625, 0.75	Buprenorphine (0.05 mg/kg) (4 h, 12 h, ql2h onwards)	Ringer’s (15 mL/kg) (12 h, ql2h onwards)	Imipenem (25 mg/kg) (12 h, q12h onwards)
4	2	2020	0.75	Buprenorphine (0.05 mg/kg) (4 h, 12 h, ql2h onwards)	Ringer’s (15 mL/kg) (12 h, ql2h onwards)	Imipenem (25 mg/kg) (12 h, q12h onwards)

An outline is provided for the series of pilot studies (#1 to #4; conducted at sites 1 and 2) highlighting the dose and route of administration of the various fluids (buprenorphine, Ringer’s lactate, and antibiotics)

### Post-inoculation monitoring and endpoints

During recovery after fecal slurry or control vehicle injection, all mice were closely monitored for evidence of pain or distress (Additional file 1: Table S1), until reaching humane or study endpoint (72 h). As death is not an acceptable experimental endpoint in accordance with the CCAC ethics standards, ARRIVE guidelines 2.0, and MQTiPSS consensus, surrogate markers of mortality were employed, using criteria related to pain, suffering, and/or illness severity [2, 11, 26]. The modified Murine Sepsis Score (MSS) involves observing posture, respiration quality, responsiveness, activity, and appearance [26, 27]. The mouse grimace scale (MGS) involves the scoring of orbital tightening, nose and cheek bulge, ear positioning, and whisker change [26, 28]. The modified MSS and MGS component scores were standardized to a four-point scale ranging from 0 (healthy) to 3 (sick) to make relevant comparisons between these scoring systems (Additional file 1: Table S1). MSS and MGS appeared concordant throughout the first series of experiments (data not shown); after increasing analgesia dose in series #2 (see below), MGS was abandoned to minimize workload during experimentation. Mice were humanely euthanized if their average MSS was equal to or greater than 1.75, or if any component of MSS was equal to 3; mice were also euthanized if they reached the end of the study (72 h). Scoring was performed independently by two observers and the mean of these scores was recorded at each time-point for each component in each animal. At site 1, temperature was recorded rectally using a Harvard Apparatus Homeothermic Monitor (Harvard Apparatus Canada, Saint-Laurent, Quebec, Canada). At site 2, temperature was recorded rectally using a digital mouse thermometer BIO-TK8851 and a mouse probe BIO-BRET-3 (Bioseb Lab Instruments, France). In series #1 and #2 of experiments at site 1, temperature recordings were done under isoflurane anesthesia; the use of anesthesia for temperature measurements was discontinued due to dramatically lower body temperature recordings. In all subsequent experiments at site 1 and site 2, animals were either manually restrained or placed in a restrainer for temperature monitoring.

### Experimental design

An outline of the multiple series of experiments (series #1–4) can be found in Table 1. In series #1, our goal was to determine a dose–response of disease severity with escalating concentrations of fecal slurry inoculation. Mice were injected IP with rat fecal slurry (prepared in 2021) at a concentration range of 0.5 mg/g, 0.75 mg/g, 1 mg/g, 1.5 mg/g, or 2.5 mg/g body weight or vehicle control. Animals were administered subcutaneous injections of 200 μL of Ringer’s lactate at 4 h post-inoculation, and then 100 μL of Ringer’s lactate at 16 h and every 8 h thereafter; 0.01 mg/kg of buprenorphine was administered subcutaneously at 4 h post-inoculation, 16 h, and then every 8 h. Mice were administered an IP injection of 25 mg/kg of piperacillin–tazobactam every 8 h post-inoculation. Treatment schedule for series #1 can be found in Table 1.

In series #2, the following changes were made: all fluids were weight-adjusted, the antibiotic piperacillin/tazobactam was replaced with imipenem, and the dose of buprenorphine was increased. The change in antibiotics was intended to provide an easier dosing schedule with imipenem (q12h) as opposed to piperacillin–tazobactam (q8h); this change also modified the timing of the first dose of antibiotics from 8 to 4 h post-inoculation. Mice were injected IP with rat fecal slurry at 0.75 mg/g or vehicle control. Fluids were 20 mL/kg of Ringer’s lactate subcutaneous at 4 h, and 12 h, and then 15 mL/kg at 24 h and every 12 h. Analgesia was 0.05 mg/kg of buprenorphine subcutaneous at 4 h, 12 h, and every 12 h. Antibiotics were 25 mg/kg of imipenem IP at 4 h, 12 h, and every 12 h. Treatment schedule for series #2 can be found in Table 1.

Based on the universal survival results from series #2, the fluid and antibiotic schedule was further modified for series #3. The rationale was that delayed administration of supportive treatments would allow sufficient time for disease progression and better approximation of clinical sepsis (i.e., patients are unlikely to present to the emergency department at the immediate onset of infection). In series #3, mice received an IP injection of rat fecal slurry at 0.75 mg/g or vehicle control. Mice received IP injections of 25 mg/kg of imipenem and subcutaneous injections of 15 mL/kg of Ringer’s lactate at 12 h and then every 12 h. Buprenorphine was administered subcutaneously at 0.05 mg/kg at 4 h, 12 h, and then every 12 h. The treatment table for series #3 can be found in Table 1.

To test reproducibility within the same lab and generalizability of findings between different labs, series #3 was repeated at site 1 (one week apart), and site 2 using the same dose of fecal slurry (0.75 mg/g body weight) and treatment schedule. A dose–response experiment was also repeated at site 2 using fecal slurry doses 0.5 mg/g and 0.625 mg/g (plus 0.75 mg/g from above) using the same treatment schedule. Comparison of dose–response curves between sites was undertaken using the Hill equation [29]. Lastly, in series #4, at site 2, a different batch of fecal slurry (prepared in 2020) was used, with the treatment schedule being consistent with series #3.

### Protocol harmonization

To ensure a harmonized implementation of the protocols between sites, numerous steps were taken. Highly qualified personnel (HQP) performing the experiments were properly trained in the experimental techniques, such as injection procedures, animal handling, monitoring, and wellness checks, as well as tissue collection, via online training sessions and regular meetings with all NPSP investigators. In addition to the interactive meetings, detailed standard operating procedures as well as video training modules were also shared in a collective online repository.

### Peritoneal cavity fluid collection

When animals reached humane or experimental endpoint, mice were anesthetized with isoflurane and oxygen inhalation. Using a 27-gauge needle, 3 mL of PBS was injected into the peritoneal cavity and the fluid was collected.

### Blood collection

At humane or experimental endpoint, under isoflurane anesthesia, blood was collected via the inferior vena cava with a 23-gauge needle into a 1:10 volume of 3.2% sodium citrate for terminal exsanguination (approx. 800–1000 μL). Plasma was prepared by centrifugation at 5000 × g for 10 min (twice) and stored in 50–100 μL aliquots at − 80 °C.

### Quantitative bacterial cultures

At humane or experimental endpoint, bacterial loads were assessed in the peritoneal cavity fluid (PCF) and blood. Tenfold serial dilutions of PCF and blood in sterile phosphate buffered saline were prepared. Ten μL of each dilution (10- to 10,000-fold) was spotted in triplicate on 5% blood agar plates. The agar plates were incubated overnight and colonies from the highest dilution were counted. The CFU/mL of blood/PCF were calculated as follows: (total # colonies / 3) × (dilution) × 100.

### Microbiome characterization

Bacterial composition of fecal slurry from 2021 to 2020 batch was evaluated using 16 s rRNA gene amplicon sequencing analysis. Total DNA was isolated from thawed cecal samples using the DNeasy PowerSoil (Qiagen) following the manufacturer’s protocol. Negative control water samples were processed identically and run through the study protocol as controls. The 16 s rRNA V4 hypervariable region was amplified by PCR dual indexed primers with sample barcodes and sequencing adaptors and PCR conditions as previously described [30]. Following amplification, the size selection and PCR products cleanup was performed using Nucleomag beads (Macherey Nagel) following manufacturer’s instructions. Individual sample libraries were normalized using a SequalPrep Normalization Plate (Invitrogen), after which samples were pooled to create the final library. Following pooling, quality control was performed on the pooled NGS libraries using the Agilent Technologies 2200 TapeStation and Qubit dsDNA analyzer. The pooled 16S V4 amplicon library was sequenced using an Illumina MiSeq platform to produce 2 × 250 bp paired-end reads.

Illumina MiSeq paired-end reads (FASTQ) were then demultiplexed and processed in R v4.1.2 following the *DADA2* pipeline v1.14 [31] for data processing and analysis. Reads were truncated to 200 bp or at a quality score Q < 2. Reads containing more than 2 errors or with ambiguous nucleotides (N) were removed. Taxonomy of unique ASVs was assigned in *DADA2* by the RDP Classifier using the SILVA v138.1 database. Contaminants were identified and removed by the *Decontam* package v.1.14.0 using negative control samples as references [32]. Cleaned samples with annotated ASVs and sample data were finally loaded into the *Phyloseq* package v.1.38.0 for further downstream analysis [33]. Diversity metrics such as alpha and beta-diversity (community dissimilarity) were calculated using the *Microbiome* package v.1.16.0 (microbiome.github.com/microbiome) and the *Vegan* package v.2.6, respectively [34]. Community dissimilarity (beta-diversity) was calculated on the Bray–Curtis dissimilarity measure by permutational ANOVA (PERMANOVA) using the adonis2 function in *Vegan*. Differential abundance analysis between cecal slurry batches was performed using functions contained within the *microbiomemarker* R package v.1.0.2 [35]. To address recent reports of variation among taxon detection in differential abundance analysis tools [36], we used two different tools to identify differentially abundant bacterial families, ANCOM (Analysis of Compositions of Microbiomes) [37] and LEfSe (Linear discriminant analysis Effect Size) [38], and report the consensus findings between both tools.

### Quantification of interleukin-6 and thrombin–antithrombin

Interleukin-6 (IL-6) was quantified using Mouse IL-6 Duoset ELISA (R&D Systems, Minneapolis, Minnesota, USA). Thrombin–antithrombin (TAT) complex levels was quantified using the TAT matched-pair antibody set (Affinity Biologicals, Ancaster, Ontario, Canada) according to the manufacturer’s protocol.

### Statistical analysis

Statistical analyses were performed using GraphPad Prism version 9.1.1 for Mac OS (GraphPad Software, San Diego, California, USA, www.graphpad.com). All clinical parameters (MSS, temperature, and weight) are expressed as median ± interquartile range. The association between MSS and mortality, biomarker (IL-6 and TAT) data, and bacterial load values are expressed as violin plots. Significant differences between biomarkers were determined using a one-way analysis of variance (ANOVA) followed by Tukey’s multiple comparisons test. *p*-values < 0.05 were considered significant. Survival curves were analyzed using a Log-rank (Mantel–Cox) test.

## Results

### Dose titration of fecal slurry shows a critical threshold for disease severity, with good reproducibility between sites despite site-dependent variability

**Fig. 1:**
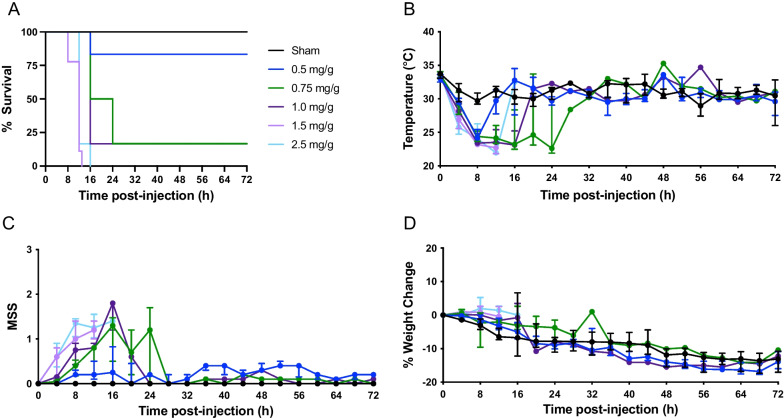
Dose titration of fecal slurry shows a threshold disease severity effect at site 1. Kaplan–Meier survival curves (**A**), temperature (**B**), MSS (**C**), and percent weight change (**D**) over time in FIP-treated and sham-treated mice at site 1. See series 1 for experimental details (Table 1). Data are presented as median ± interquartile range for sham-treated mice (*n* = 8; 4 male, 4 female), 0.5 mg/g fecal slurry (*n* = 6; 3 male, 3 female), 0.75 mg/g fecal slurry (*n* = 6; 3 male, 3 female), 1.0 mg/g fecal slurry (*n* = 6; 3 male, 3 female), 1.5 mg/g fecal slurry (*n* = 9; 5 male, 4 female), and 2.5 mg/g fecal slurry (*n* = 6; 3 male, 3 female)

In series #1 at site 1, a dose–escalation experiment was performed to assess the impact of ascending concentrations of fecal slurry inoculation on disease severity. Mortality was 0% in the sham-treated mice (*n* = 8; 4 male, 4 female), and in FIP mice mortality was 17% for 0.5 mg/g fecal slurry (*n* = 6; 3 male, 3 female), 83% for 0.75 mg/g fecal slurry (*n* = 6; 3 male, 3 female), 83% for 1.0 mg/g fecal slurry (*n* = 6; 3 male, 3 female), 100% for 1.5 mg/g fecal slurry (*n* = 9; 5 male, 4 female), and 100% for 2.5 mg/g fecal slurry (*n* = 6; 3 male, 3 female) (Fig. 1A). No sex difference was observed for mice administered varying doses of fecal slurry (Additional file 1: Figure S1). All FIP mice experienced a drop in temperature at 4 h post-inoculation (Fig. 1B). Mice subjected to high concentrations of fecal slurry had higher MSS scores within 16 h compared to mice administered low concentrations of fecal slurry (Fig. 1C). All mice exhibited a weight loss or gain of approximately 20% or less from baseline throughout the course of the experiment (Fig. 1D). Based on our dose-titration studies, we observed a critical threshold, where there was either severe (> 60%) or minimal (< 20%) mortality in the model depending on the dose of fecal slurry.

**Fig. 2:**
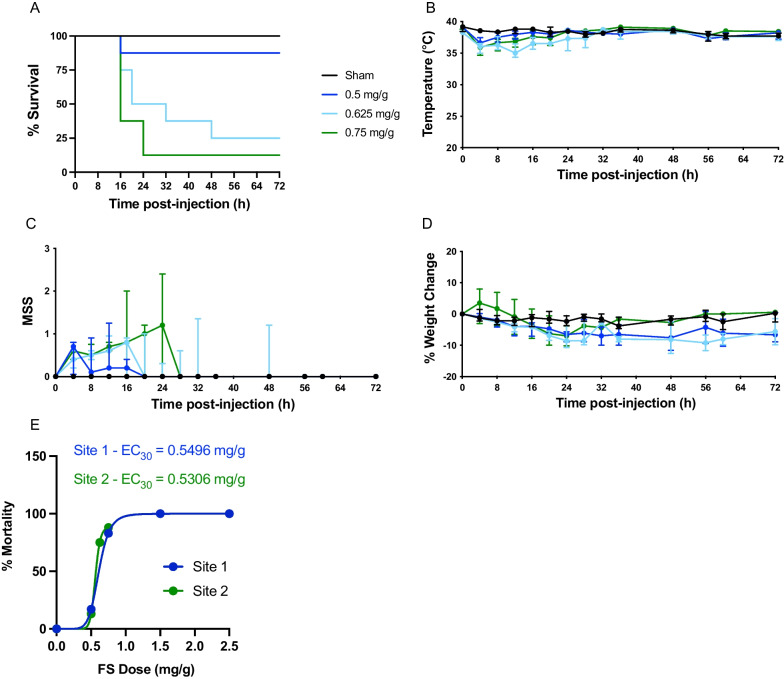
Dose titration of fecal slurry shows a threshold disease severity effect at site 2. Kaplan–Meier survival curves (**A**), temperature (**B**), MSS (**C**), percent weight change (**D**), and Hill equation (**E**) over time in FIP-treated and sham-treated mice at site 2. See series 3 for experimental details (Table 1). Data are presented as median ± interquartile range from sham-treated mice (*n* = 6; 3 male, 3 female), 0.5 mg/g fecal slurry (*n* = 8; 4 male, 4 female), 0.625 mg/g fecal slurry (*n* = 8; 4 male, 4 female), and 0.75 mg/g fecal slurry (*n* = 8; 4 male, 4 female)

A dose escalation study was also conducted at site 2 (series #3), as described in the methods. Compared to sham-treated mice (*n* = 6; 3 male, 3 female), which had a mortality rate of 0%, FIP mice had a mortality rate of 13% for 0.5 mg/g of fecal slurry (*n* = 8; 4 male, 4 female), 75% for 0.625 mg/g of fecal slurry (*n* = 8; 4 male, 4 female), and 88% for 0.75 mg/g fecal slurry (*n* = 8; 4 male, 4 female) (Fig. 2A). A sex difference was observed for mice administered 0.75 mg/g of fecal slurry, with males demonstrating increased mortality (Additional file 1: Figure S2C). All FIP mice experienced a decrease in body temperature at 4 h post-inoculation compared to sham mice (Fig. 2B). Mice administered fecal slurry resulted in high MSS scores until 24 h post-inoculation (Fig. 2C). All mice exhibited a weight loss or gain of approximately 10% from baseline throughout the course of the experiment (Fig. 1D). Furthermore, as demonstrated by the Hill equation, the dose–response studies conducted at site 1 and site 2 show comparable mortality curves (Fig. 2E); the estimated dose concentration to achieve 30% mortality was 0.5496 mg/g and 0.5306 mg/g for site 1 and site 2, respectively.

**Fig. 3:**
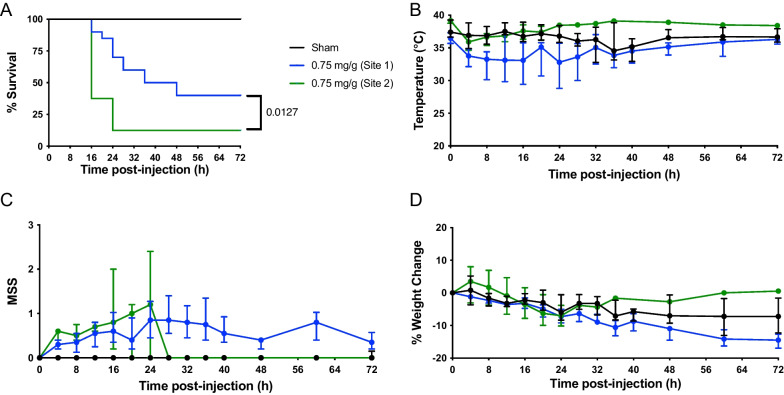
Reproducibility of fecal-induced peritonitis model between sites. Kaplan–Meier survival curves (**A**), temperature (**B**), MSS (**C**), and percent weight change (**D**) over time in FIP-treated and sham-treated mice. Data are presented as median ± interquartile range from FIP mice with 0.75 mg/g at site 1 (*n* = 20; 10 male and 10 female) and FIP mice with 0.75 mg/g at site 2 (*n* = 8; 4 male and 4 female); sham animals were pooled between both sites (*n* = 6; 3 males and 3 females). See series 3 at site 1 and site 2 for experimental details (Table 1). Survival curves between site 1 and site 2 were analyzed using a Log-rank (Mantel–Cox) test; *p*-values < 0.05 were considered significant

To assess the variability in outcomes of our FIP model between laboratories, we compared 0.75 mg/g fecal slurry at site 1 (*n* = 20; 10 male, 10 female divided in two experiments) and site 2 (*n* = 8; 4 male, 4 female) using the same antibiotic and treatment schedule (series #3). Both experiments at site 1 had identical mortality and the results were pooled for analysis. As shown in Fig. 3A, there was a significant (*p* < 0.05) difference in mortality between site 1 (60%) and site 2 (88%). FIP mice demonstrated a decrease in body temperature at 4 h, with temperatures returning to baseline at experimental endpoint (Fig. 3B). Furthermore, the temperature of the mice was different between the two sites, with site 1 having consistently lower temperatures for the animals throughout the experiment compared to stable temperature recordings for site 2. FIP mice at both sites exhibited increased MSS scores between 4 and 24 h (Fig. 3C). Differences in body weight changes were observed between sites: FIP mice at site 2 returned to baseline weight at approximately 36 h, whereas FIP mice at site 1 exhibited a continuous decrease in weight throughout the study (Fig. 3D). A sex difference was observed for mice at site 2, with males demonstrating increased mortality (Additional file 1: Figure S3B).

Together, these experiments suggest that many findings of the FIP model were reproducible within each lab and between labs using standardized protocol. However, site-dependent variability in mortality, temperature, and weight change reinforces the added complexity introduced by a multi-laboratory format.

### The Murine Sepsis Score (MSS) can assess disease severity, but is not reliably associated with death

**Fig. 4:**
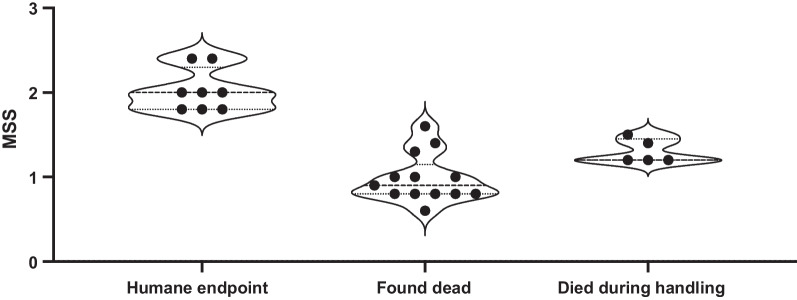
The Murine Sepsis Score (MSS) is not reliably associated with death. MSS for mice that either reached humane endpoint (*n* = 8), were found dead (*n* = 13), or died during handling (*n* = 5). Animals received FIP with 0.75 mg/g; see series 3 for experimental details at site 1 and site 2 (Table 1). Data are presented as a violin plot. MSS for animals found dead was within acceptable range for ongoing experimentation

The modified MSS system was useful in assessing the severity of disease as higher doses of fecal slurry inoculation resulted in higher MSS over time (Figs. 1C and 2C). However, modified MSS was not associated with death in our FIP model of sepsis. During the 72-h study period, 8/26 mice reached the humane endpoint (average MSS ≥ 1.75 or MSS component = 3), 13/26 mice were found dead, and 5/26 mice died during handling (Fig. 4). The median MSS for mice that were culled, were found dead, and died during handling was 2.0, 0.9, and 1.2, respectively. Together, these observations suggest that although higher MSS scores are associated with increasing disease severity in alive animals, low scores do not preclude rapid progression to death in our murine model of sepsis.

### Early therapeutic intervention with antibiotics rescues animals from sepsis and reduces bacterial load

**Fig. 5:**
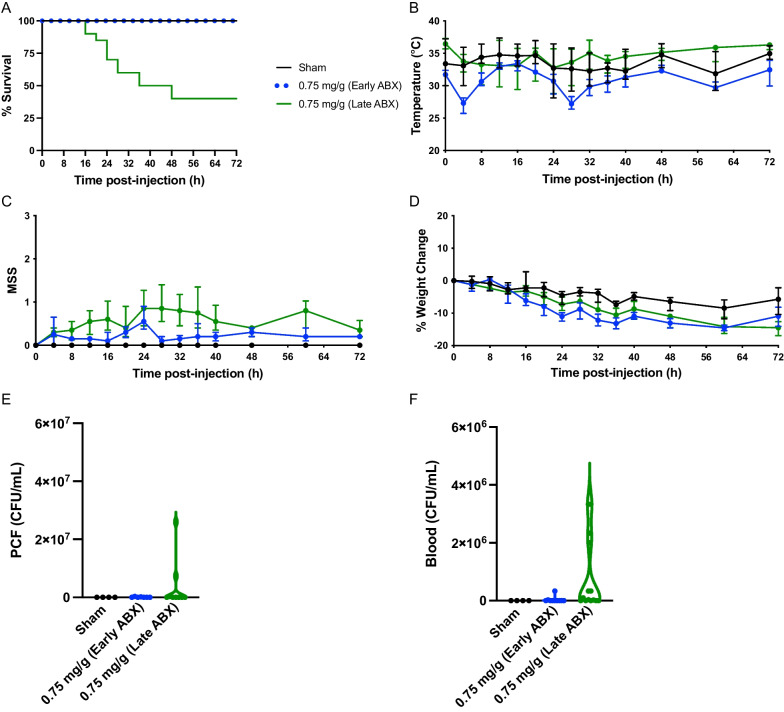
Timing of antibiotic administration shows effect on disease severity. Kaplan–Meier survival curves (**A**), temperature (**B**), MSS (**C**), and percent weight change (**D**) over time in FIP-treated and sham-treated mice. Animals received FIP with 0.75 mg/g; see series 2 (early) and series 3 (late) at site 1 for experimental details (Table 1). Data are presented as median ± interquartile range from sham-treated mice (*n* = 8; 4 male, 4 female), FIP-treated mice with early intervention (*n* = 10; 5 male, 5 female), and FIP-treated mice with late intervention (*n* = 20; 10 male, 10 female). Bacterial loads for PCF (**E**), and bacterial loads for blood (**F**) were recorded for each mouse. Data are presented as violin plots. Confluent bacterial loads were omitted as the colonies were uncountable

Next, we aimed to determine if the timing of antibiotics and fluids impacts mortality in our FIP model. Mice were injected with a fecal slurry dose of 0.75 mg/g body weight and received either early intervention with antibiotics (imipenem) and fluid resuscitation (4 h, 12 h, and every 12 h) or late intervention with antibiotics (imipenem) and fluid resuscitation (12 h, and every 12 h). As shown in Fig. 5A, early intervention rescued all mice (*n* = 10; *n* = 5 for male and female) with no observed mortality. In comparison, late intervention with delayed fluid and antibiotics at 12 h resulted in 60% mortality (*n* = 20; *n* = 10 for male and female). No sex difference was observed between the early intervention group and late intervention group (Additional file 1: Figure S4). Both groups exhibited decreased body temperature at 4 h, with the temperature returning to baseline at experimental endpoint; interestingly, the early antibiotic group had a more pronounced drop in body temperature despite having increased survival (Fig. 5B). Compared to mice which received early antibiotics, mice which received late antibiotics had higher MSS beginning at 12 h until endpoint (Fig. 5C). All mice exhibited a change in weight from baseline, with the FIP mice experiencing a greater weight change compared to the sham mice (Fig. 5D). These survival differences between groups correlated with bacterial loads in the PCF and blood (collected at humane or experimental time-point), that were increased in the late antibiotic group compared to the early intervention group (Fig. 5E and F). Furthermore, out of 10 mice that received early antibiotics, 3 mice exhibited confluent bacterial loads for PCF (not shown in Fig. 5E and F as colonies were uncountable). Out of 20 mice that received late antibiotics, 11 and 5 mice exhibited confluent bacterial loads for PCF and blood, respectively (not shown in Fig. 5E and F as colonies were uncountable).

Results from these imipenem studies can also be compared with data from series #1, which used piperacillin–tazobactam (8 h, then every 8 h) and fluids (4 h, 16 h, then every 8 h). The timing for the antibiotic regimen from series #1 was intermediate—between early (4 h) and late (12 h), whereas the timing of fluid administration was early (4 h). The mortality for 0.75 mg/g fecal slurry in series #1 was 83%—comparable to the mortality in the late antibiotic group with imipenem (60%), and much higher than the mortality in the early antibiotic group (0%). Overall, these results suggest that delayed administration of antibiotics results in a severe model compared to early administration of antibiotics that rescues animals from sepsis and prevents bacterial load in the PCF and blood; early fluid resuscitation alone appears to have minimal effects on disease severity if not combined with early antibiotics.

### Differential septic response to inoculum and microbial composition between fecal slurry batches

**Fig. 6:**
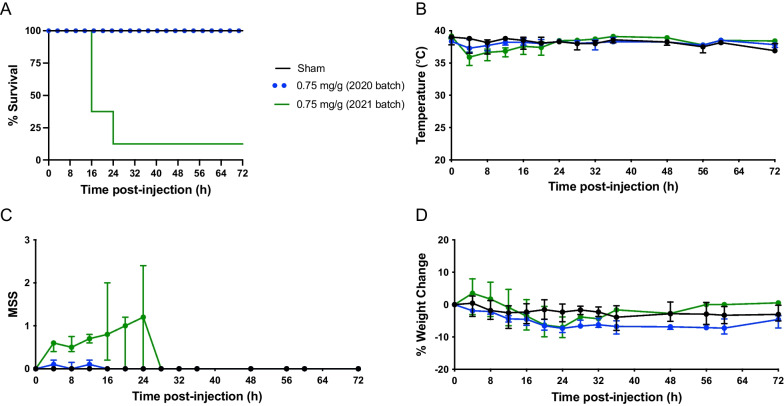
Disease severity in FIP model is dependent on fecal slurry batch. Kaplan–Meier survival curves (**A**), temperature (**B**), MSS (**C**), and percent weight change (**D**) over time in FIP-treated and sham-treated mice. Animals received FIP with 0.75 mg/g; see series 3 (2021) and series 4 (2020) at site 2 for experimental details (Table 1). Data are presented as median ± interquartile range from sham-treated mice (*n* = 4; 2 male, 2 female), FIP-treated mice with 2020 batch (*n* = 8; 4 male, 4 female), and FIP-treated mice with 2021 batch (*n* = 8; 4 male, 4 female)

To assess variation between fecal slurry batches on disease outcome, mice were injected IP with 0.75 mg/g body weight of fecal slurry prepared in either the 2020 batch or the 2021 batch; antibiotics (imipenem) and fluid timing were the same in these experiments. As shown in Fig. 6A, mice administered 2020 batch of slurry had 0% mortality (*n* = 8, *n* = 4 for male and female), whereas mice administered 2021 batch of slurry had 88% mortality (*n* = 8, *n* = 4 for male and female). A sex difference was observed for mice administered 2021 batch of fecal slurry, with males demonstrating increased mortality (Additional file 1: Figure S5B). FIP mice demonstrated a drop in body temperature 4 h post-inoculation (Fig. 6B). Mice administered the 2021 batch had elevated MSS compared with sham mice and mice treated with the 2020 batch of slurry (Fig. 6C). Mice administered the 2020 batch exhibited a steady decrease in weight, whereas mice administered 2021 batch of the slurry exhibited a transient decrease in weight, followed by an increase (Fig. 6D).

**Fig. 7:**
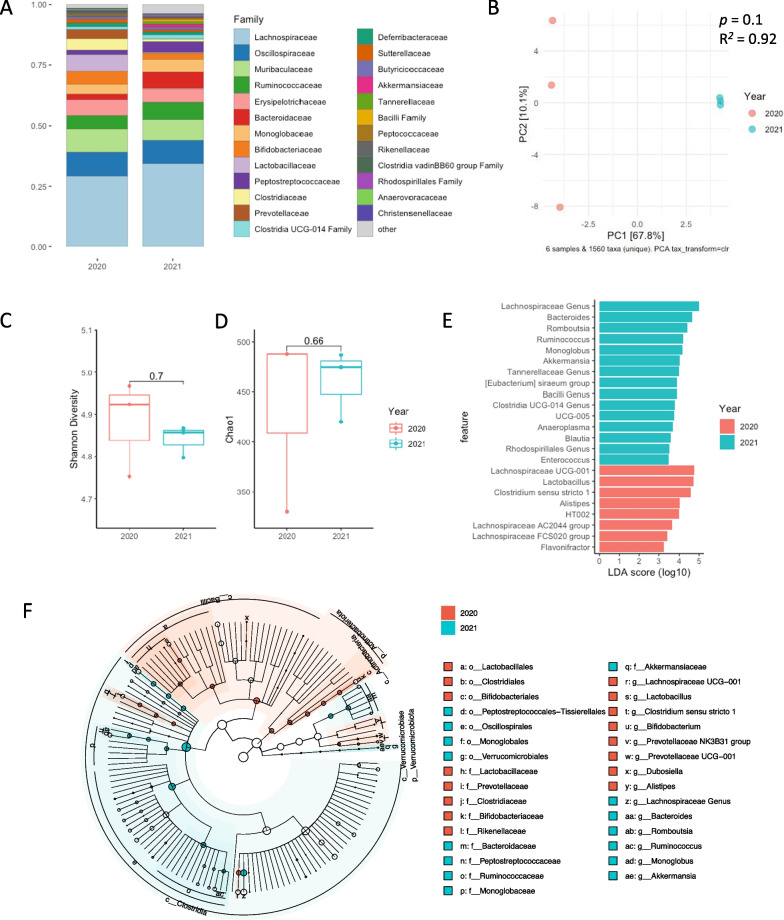
Differential septic response to inoculum between fecal slurry batches. The microbiota composition of the fecal slurry batches from 2020 (*n* = 3) and 2021 (*n* = 3) was determined using 16S rRNA amplicon sequencing. Bar plots depict the relative abundance of the top 25 bacterial genera from the 2020 batch and the 2021 batch (**A**). Principal coordinate analysis (PCoA) of the fecal slurry microbiota was calculated from the Bray–Curtis dissimilarity distance of CLR transformed ASVs (**B**). Statistical significance was determined using a permutational ANOVA (PERMANOVA). *p* values as shown. Taxonomic diversity as represented by **C** Shannon diversity and **D** Chao1 was calculated for individual fecal slurry samples at the ASV level and differences between the 2020 batch and 2021 batch were determined using a Wilcoxon test, *p* values as shown. Differential abundance analysis was performed using linear discriminant analysis effect size (LEfSe) and analysis of composition of microbiomes (ANCOM) to determine differentially abundant bacterial genera between the 2020 batch and the 2021 batch (**E**). Bacterial genera identified by both tools to be differentially abundant are shown here. A cladogram depiction of the bacterial taxa identified as differentially abundant between the 2020 fecal slurry batch and 2021 fecal slurry batch by LEfSe (**F**)

Next, to determine if the difference in survival between the fecal slurry batches may be related to differential bacterial composition, we performed metagenomic assessment of the bacterial microbiome composition of multiple aliquots of each fecal slurry batch using 16 s rRNA gene amplicon sequencing. The distribution of bacterial taxa differed between batches of fecal slurry, as did bacterial community β-diversity demonstrated by separation of the batches on principal component analysis (Fig. 7A, B). Of note, diversity and richness (i.e., number of unique amplicon sequence variants) of bacteria were similar between slurry batches (Fig. 7C, D). To determine whether there were particular bacterial families driving the differences, we performed a differential abundance analysis that revealed 23 differentially abundant taxa across a diverse spectrum of bacterial families, with 8 enriched genera in the 2020 batch and 15 in the 2021 batch (Fig. 7E, F). A number of potentially pathogenic organisms were differentially represented including *Bacteroidaceae* and *Enterococcaceae* in the 2021 batch and *Prevotellaceae* in the 2020 batch, along with multiple taxa that are typically non-pathogenic (*Ruminococcaceae*, *Tannerellaceae*, *Lactobacillaceae*, Fig. 7E).

### Septic non-survivors exhibit elevated levels of inflammation and coagulation

**Fig. 8:**
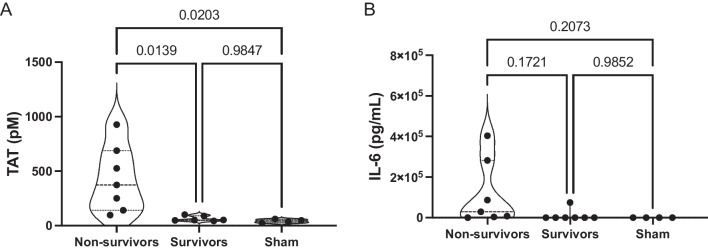
Septic non-survivor mice exhibit elevated levels of coagulation and inflammation. Plasma levels of TAT (**A**) and IL-6 (**B**) were quantified and compared to levels observed in septic non-survivors, septic survivors, and sham mice. Data are presented as violin plots representing sham mice (*n* = 4), septic non-survivors (*n* = 7), and septic survivors (*n* = 6–7). Statistical significance was determined using a one-way ANOVA. *p*-values < 0.05 were considered significant

To examine the changes in markers of coagulation (TAT) and inflammation (IL-6) in a murine model of FIP induced sepsis, mice were inoculated with 0.75 mg/g of fecal slurry or control vehicle and euthanized upon reaching humane or experimental endpoint (72 h). As shown in Fig. 8A, septic non-survivors exhibited elevated levels of TAT compared to septic survivors, as well as to sham mice. As shown in Fig. 8B, there was no significant difference in IL-6 levels between septic non-survivors and septic survivors. Both TAT and IL-6 levels demonstrated a high degree of variability in septic non-survivors, which was not observed in septic survivors and sham animals.

## Discussion

To overcome methodological shortcomings of preclinical sepsis research, we created the NPSP, a collaborative multi-laboratory network of Canadian basic science sepsis researchers and veterinarians [1]. We aimed to establish a standardized and reproducible 72-h model of polymicrobial abdominal sepsis using FIP. Through a series of iterative studies conducted at two experimental sites, we have robustly characterized our model, gained insight into how individual experimental variables affect model outcomes, and highlighted important considerations for multi-laboratory preclinical sepsis research.

In this current study, we found that ascending concentrations of fecal slurry inoculation resulted in a threshold disease severity effect. As shown in Figs. 1A and 2A, a concentration of fecal slurry inoculum at 0.5 mg/g resulted in minimal mortality (< 20%), whereas a concentration of fecal slurry inoculum equal to or greater than 0.625 mg/g resulted in severe mortality (> 60%). Decreased temperature and increased MSS reflect the clinical profiles of mice subjected to increasing concentrations of fecal slurry (Figs. 1B, C and 2B, C). Our findings are consistent with previous studies demonstrating a dose-dependent increase in mortality and MSS in murine models of FIP with increasing concentrations of fecal slurry [27, 39]. Due to the bimodal distribution of mortality with this model, a moderate mortality approximation of 30–40% was not achieved. The sigmoidal dose–mortality relationship shown in Fig. 2E suggests even modest changes in slurry dosing can markedly affect disease severity. Indeed, whereas a slurry dose of approximately 0.54 mg/g (as calculated by the Hill equation) would be expected to generate a mortality rate of 30%, the dose of 0.625 mg/g appears to induce an illness above our intended disease threshold, resulting in a rapid clinical deterioration. This may be attributed to small numbers of animals used in these experiments, or the vulnerable physiological status of the mice, whereby they appear to reach a “tipping point” and then experience a rapid clinical deterioration. Furthermore, some sex-related differences were observed in our study (Additional file 1: Figures S1-S5), with males exhibiting a greater mortality than females, consistent with previous sepsis studies [4]; given the small sample sizes, these results should be viewed as preliminary and require further testing in adequately powered studies.

To ensure complete experimental standardization between sites, the following steps were taken: full harmonization of protocols, technical training, sharing of detailed standard operating procedures, and creation of video training modules. This multi-laboratory format represents a significant advancement in the field of preclinical research. However, as shown in Fig. 3A, there was a significant difference in mortality rate between site 1 (60%) and site 2 (88%) for the same dose of fecal slurry and treatment schedule. Possible reasons for this difference in survival include a small sample size of mice, environmental conditions (e.g., housing and lab microbiome), different operators, biological variability of the animals, and biological heterogeneity of sepsis. As shown in Fig. 3B, septic mice at site 1 exhibited lower temperatures (approximately 27–38 °C), compared to septic mice at site 2, (approximately 34–39 °C). Furthermore, modifications to the method for temperature collection (with or without isoflurane anesthesia) had dramatic impacts on core body temperature at site 1 (Fig. 1B vs Fig. 3B). Past studies have shown that septic mice with higher core body temperatures display increased mortality rate [40, 41], and that temperature is an often-overlooked factor in preclinical sepsis models that impacts reproducibility and variability [41]. Similarly, despite animals at site 1 having lower body temperatures, their survival was actually higher than site 2. Therefore, future multi-laboratory studies should tightly regulate and control the ambient temperature, animal warming, and temperature collection techniques to reduce any potential bias and heterogeneity in the results. Yet, the presence of survival differences between sites also reinforces the need and value of the NPSP multi-laboratory format which accounts for outcome variability between centers; for example, the impact of positive findings is strengthened if they are observed in multiple laboratories.

According to the CCAC ethical guidelines, which precludes death as an acceptable endpoint, mice were regularly monitored until they reached a pre-specified humane endpoint that is used as a surrogate marker of mortality. The modified MSS system is used in our study to evaluate the clinical condition of mice with experimental sepsis across multiple components, including posture, appearance, activity, response to stimulus, and respiratory quality. Due to the potentially subjective nature of this scoring system, body temperature and weight were also recorded as objective physiological measures. Mice administered higher doses of fecal slurry inoculation resulted in higher MSS over time, demonstrating that the MSS is effective in determining disease progression (Figs. 1C and 2C). However, over time, MSS became an increasingly unreliable surrogate for disease progression and death in these animals (Fig. 4); indeed, the score of animals that were found dead was within the acceptable clinical range for continued experimentation. In contrast to Shrum et al.’s findings [27], where the MSS system displays a high specificity and sensitivity in predicting mortality in a FIP model of sepsis, our findings suggest that the scoring system was more variably associated with death. One possible explanation for this discrepancy is the difference in experimental design of the models. In our model of sepsis, the use of fluid resuscitation and antibiotics were incorporated, whereas in Shrum et al.’s study, fluid management was omitted [27]. Thus, it is possible that resuscitation and antibiotics modifies the untreated progression of disease severity.

These results raise important questions regarding the use of clinical scoring systems of disease severity and/or mortality in animal models of sepsis. Firstly, these scores should be carefully re-evaluated when experimental modifications are implemented, and comparison between different models may not always be appropriate. Secondly, it seems reasonable to assume there are biological difference between animals found dead versus those that meet a surrogate endpoint criteria based on a quasi-subjective (albeit standardized) assessment. By grouping both of these types of animals into the “mortality” cohort, we are introducing heterogeneity and bias into any outcome evaluation that follows (e.g., site-specific mortality differences). Moreover, animals that remain in the “survivor” cohort may all be subject to selection bias. Lastly, because animals will exit the study at different timepoints due to mortality, our ability to group them together and compare their biology to survivor animals that complete the entire study duration may not be appropriate. This is demonstrated with our biomarker data, whereby non-survivors exhibited high degrees of variability in measurements, and survivors all appear to have normalized levels of IL-6 and TAT at 72 h (Fig. 8A, B). Thus, we conclude that using surrogate mortality outcomes poses significant challenges for external validity of preclinical sepsis models. However, we recognize the need for incorporating relevant outcomes that are meaningful to clinicians and patients; this may be a tension that is not easily resolved. In order to overcome these obstacles, it may be reasonable to design models with no anticipated mortality, to limit bias in outcomes and analysis and focus exclusively on disease pathogenesis. Because the goal of this current study was model development, our primary reported outcomes were general markers of disease severity (e.g., MSS). Future work with this model will explore mechanistic pathways and organ injury with analysis of plasma biomarkers and histology. This approach will maximize the translational utility of preclinical sepsis models while maintaining their fidelity to experimental design and intended purpose.

To mimic the clinical situation, routine supportive therapies including antibiotics and fluid resuscitation are vital to incorporate into preclinical models [1, 2, 7]. Based on our findings from series #2 of 0% mortality, and the observation that septic patients seldom present to the ER at the immediate onset of sepsis, we subsequently delayed antibiotic administration to 12 h post-FIP injection. As shown in Fig. 5A, E, F, delayed administration of antibiotics at 12 h results in a severe model (60% mortality) as well as high bacterial loads in the PCF and blood compared to early administration of antibiotics that rescues animals from sepsis. These findings agree with Steele and colleagues, who showed that late administration of antibiotics and fluids (12 h or 24 h) resulted in a longer disease course and downstream pathology, while maintaining a high survival rate [42]. Their model similarly allows for the progression of bacteremia which becomes evident at later points (12 h or 24 h) and more closely mimics the pathological characteristics of sepsis [42].

Our study also demonstrated that variability in microbial composition of fecal slurry is an important contributor to disease severity and outcomes in preclinical sepsis models (Figs. 6 and 7). Notably, this variability was present despite best efforts to standardization of slurry preparation. Our data revealed that differences in bacterial composition were primarily among anaerobic fermenters, including several potentially pathogenic organisms. While this may reflect intrinsic differences in the gut microbiome between donor animals, given that many differentially abundant organisms were anaerobic fermenters, this raises the question of whether subtle differences in slurry preparation (e.g., duration of ambient oxygen exposure in its impact on obligate anaerobes) may yield important differences in bacterial composition, translating into differences in disease severity in the FIP model. These findings highlight the variability in outcomes that can be induced by using different slurry batches within an experiment, and therefore for large multi-center experiments, supports the use of single large batch preparations of slurry and a need for re-calibration and dose-titration with each new batch of fecal slurry. Although it is possible that duration of storage impacted the virulence of our slurry, all experiments conducted with the 2021 batch over the course of multiple experimental series appear to be concordant in terms of their outcomes. To our knowledge, few preclinical studies routinely characterize the microbial composition of fecal slurry, and reporting of this data is expected to enhance experimental transparency and rigor. Our findings also highlight the potential impact of fecal microbial composition in preclinical sepsis outcomes, as has been shown by other groups [39].

This study has some limitations that should be acknowledged. Each experimental series included a small number of animals per group, which were not adequately powered to detect differences for many of our clinical outcomes (e.g., mortality, temperature, MSS, and sex differences); similarly, minimal statistical calculations in this study should be interpreted cautiously in this exploratory study with no power calculations. Although rigorous standardization was attempted between sites, differences in ambient temperature persisted between animal care facilities. Also, the experiments were not run on the same days, nor were the animals purchased from the same litter, and seasonal variability in murine outcomes cannot be excluded. Lastly, the 72-h model requires around-the-clock animal checks throughout the duration of the experiment, which was noted to be particularly labor-intensive for personnel.

In conclusion, the NPSP has developed and characterized a 72-h murine model of abdominal sepsis that can be used to investigate the pathophysiology of sepsis as well as to explore novel therapeutics. We have demonstrated how each individual component of the experimental protocol can have a large impact on outcomes, and how the use of surrogate mortality endpoints in preclinical sepsis presents unique challenges that should be carefully considered. This platform represents an important resource for maximizing translational impact of preclinical sepsis research.

## Supplementary Information

**Additional file 1.** Supplementary tables and figures.

**Additional file 2. Appendix 1:** ARRIVE guidelines checklist.

**Additional file 3. Appendix 2:** Experimental datasets

## Data Availability

Experimental datasets included in Additional file 3: Appendix 2. Further data available upon reasonable request.

## Abbreviations

ANCOM: Analysis of compositions of microbiomes
ANOVA: Analysis of variance
CCAC: Canadian Council for Animal Care
FIP: Fecal-induced peritonitis
HQP: Highly qualified personnel
IL: Interleukin
IP: Intraperitoneal
LEfSe: Linear discriminant analysis effect size
MGS: Mouse grimace scale
MSS: Murine Sepsis Score
MQTiPSS: Minimum quality threshold in preclinical sepsis studies
NPSP: National Preclinical Sepsis Platform
N: Nucleotides
PCF: Peritoneal cavity fluid
PERMANOVA: Permutational analysis of variance
TAT: Thrombin–antithrombin
TaARI: Thrombosis and Atherosclerosis Research Institute
