# Supplementary material for: Development and characterization of a fecal-induced peritonitis model of murine sepsis: results from a multi-laboratory study and iterative modification of experimental conditions
Source: Intensive Care Med Exp. 2023 Jul 17;11:45. doi: 10.1186/s40635-023-00533-3 (PMC10352196; doi:10.1186/s40635-023-00533-3)
Supplement: Supplementary file 1 — Additional file 1. Supplementary tables and figures. [file 40635_2023_533_MOESM1_ESM.docx]

**SUPPLEMENTARY MATERIAL:**

|  | Score | | | |
| --- | --- | --- | --- | --- |
|  | **0** | **1** | **2** | **3** |
| Murine Sepsis Score (MSS) | | | | |
| Posture | Slim body | Hunched slightly | Hunched | Hunched huge |
| Activity | Normal  10 + steps | Reduced  <10 steps | Little (even when provoked)  1 – 2 steps | Stationary (even when provoked)  0 steps |
| Respiration Quality | Normal | Periods of labored (slow/fast) breathing | Consistently labored (slow/fast) breathing | Labored breathing with gaps  (slow/fast) breathing |
| Responsiveness | Normal; Fast response to auditory or touch stimuli | Slowed response to auditory or touch stimuli | No response to auditory, slowed response to touch | No response to touch stimuli |
| Ruffled Fur | Normal coat; Smooth fur | Slightly ruffled fur (25% of body) | Majority of fur is ruffled (50-75% of body) | Ruffled fur and piloerection  (100% of body) |
| Mouse Grimace Scale (MGS) | | | | |
| Orbital Tightening | Eyes open | Eyes slightly closed | Eyes half closed | Eyes closed |
| Nose and Cheek Bulge | Normal, flat | Slightly rounded extension of skin around nose bridge | Wrinkled nose or checks, slight bulge in checks | Rigid appearing nose and cheek bulge |
| Ear Positioning-Erected | Ears flat, back against body (roughly perpendicular to the head and are directed forward) | Ears alert, slightly angled from back  *In pain, the ears tend to rotate outwards and/or back, away from the face. As a result, the space between the ears may appear wider relative to baseline. | Ears partially positioned forward or apart | Ears completely erect, far apart |
| Whisker-Erected | Normal, Whiskers moving | Some whiskers erect; Whiskers moving | Whiskers mostly erect or clumping; Whiskers moving slowly | All whiskers standing on end; Whiskers not moving |

**Supplementary Table 1: Endpoint Monitoring Description.**

Endpoint monitoring was used during the post-operative period to assess disease severity via the Murine Sepsis Score and pain via the Murine Grimace Scale. Components are given a score from 0 to 3 (0 representing characteristics similar to those in healthy control mice and 3 being the most severe) [1–4]. Scores were recorded on the data collection form every 4 hours until the animals reached humane endpoint or the end of the 72-hour experiment.

**Supplementary Figure 1: Sex-related differences in dose titration of fecal slurry at site 1.**

Kaplan-Meier survival curves (A-E) over time in male and female FIP-treated mice at site 1; 0.5 mg/g fecal slurry (n = 6; 3 male, 3 female), 0.75 mg/g fecal slurry (n = 6; 3 male, 3 female), 1.0 mg/g fecal slurry (n = 6; 3 male, 3 female), 1.5 mg/g fecal slurry (n = 9; 5 male, 4 female), and 2.5 mg/g fecal slurry (n = 6; 3 male, 3 female). See series 1 for experimental details (Table 1). Statistical significance was determined using a log-rank (Mantel-Cox test). P-values < 0.05 were considered significant.

**Supplementary Figure 2: Sex-related differences in dose titration of fecal slurry at site 2.**

Kaplan-Meier survival curves (A-C) over time in male and female FIP-treated mice at site 2; 0.5 mg/g fecal slurry (n = 8; 4 male, 4 female), 0.625 mg/g fecal slurry (n = 8; 4 male, 4 female), and 0.75 mg/g fecal slurry (n = 8; 4 male, 4 female). See series 3 for experimental details (Table 1). Statistical significance was determined using a log-rank (Mantel-Cox test). P-values < 0.05 were considered significant.

**Supplementary Figure 3: Sex-related differences in reproducibility of fecal induced peritonitis model between sites.**

Kaplan-Meier survival curves (A-B) over time in male and female FIP-treated mice at site 1 and site 2; 0.75 mg/g at site 1 (n = 20; 10 male and 10 female) and FIP mice with 0.75 mg/g at site 2 (n = 8; 4 male and 4 female). See series 3 for experimental details at site 1 and site 2 (Table 1). Statistical significance was determined using a log-rank (Mantel-Cox test). P-values < 0.05 were considered significant.

** Supplementary Figure 4: Sex-related differences in timing of antibiotic administration.**

Kaplan-Meier survival curves (A-B) over time in male and female FIP-treated mice administered early antibiotics and late antibiotics; early intervention (n = 10; 5 male, 5 female), and FIP-treated mice late intervention (n = 20; 10 male, 10 female). See series 2 (early) and series 3 (late) at site 1 for experimental details (Statistical significance was determined using a log-rank (Mantel-Cox test). P-values < 0.05 were considered significant.

** Supplementary Figure 5: Sex-related differences in disease severity based on fecal slurry batch.**

Kaplan-Meier survival curves (A-B) over time in male and female FIP-treated mice administered two separate batches of fecal slurry: 2020 batch (n = 8; 4 male, 4 female), and 2021 batch (n = 8; 4 male, 4 female). See series 3 (2021) and series 4 (2020) at site 2 for experimental details (Table 1). Statistical significance was determined using a log-rank (Mantel-Cox test). P-values < 0.05 was considered significant.

**REFERENCES:**

1. Sevcik MA, Jonas BM, Lindsay TH, et al (2006) Endogenous opioids inhibit early-stage pancreatic pain in a mouse model of pancreatic cancer. Gastroenterology 131:900–910. https://doi.org/10.1053/J.GASTRO.2006.06.021

2. Mai SHC, Sharma N, Kwong AC, et al (2018) Body temperature and mouse scoring systems as surrogate markers of death in cecal ligation and puncture sepsis. Intensive Care Med Exp 6:1–14. https://doi.org/10.1186/s40635-018-0184-3

3. Langford DJ, Bailey AL, Chanda ML, et al (2010) Coding of facial expressions of pain in the laboratory mouse. Nat Methods 7:447–449. https://doi.org/10.1038/NMETH.1455

4. Shrum B, Anantha R V, Xu SX, et al (2014) A robust scoring system to evaluate sepsis severity in an animal model. BMC Res Notes 7:1–11. https://doi.org/10.1186/1756-0500-7-233
