# Supplementary material for: Development and characterization of a fecal-induced peritonitis model of murine sepsis: results from a multi-laboratory study and iterative modification of experimental conditions
Source: Intensive Care Med Exp. 2023 Jul 17;11:45. doi: 10.1186/s40635-023-00533-3 (PMC10352196; doi:10.1186/s40635-023-00533-3)
Supplement: Supplementary file 3 — Additional file 3. Appendix 2: Experimental datasets [file 40635_2023_533_MOESM3_ESM.pdf]

Series #1 - Site 1: Fecal Slurry Batch 2021			
**Sex**	**Treatment group**	**Time of death (h)**	
M	Fecal slurry 0.5 mg/g	72	Experimental endpoint
M	Fecal slurry 0.5 mg/g	16	Humane endpoint
M	Fecal slurry 0.5 mg/g	72	Experimental endpoint
F	Fecal slurry 0.5 mg/g	72	Experimental endpoint
F	Fecal slurry 0.5 mg/g	72	Experimental endpoint
F	Fecal slurry 0.5 mg/g	72	Experimental endpoint
M	Fecal slurry 0.75 mg/g	16	Humane endpoint
M	Fecal slurry 0.75 mg/g	16	Humane endpoint
M	Fecal slurry 0.75 mg/g	72	Experimental endpoint
F	Fecal slurry 0.75 mg/g	24	Humane endpoint
F	Fecal slurry 0.75 mg/g	16	Humane endpoint
F	Fecal slurry 0.75 mg/g	24	Humane endpoint
M	Fecal slurry 1.0 mg/g	16	Humane endpoint
M	Fecal slurry 1.0 mg/g	16	Humane endpoint
M	Fecal slurry 1.0 mg/g	16	Humane endpoint
F	Fecal slurry 1.0 mg/g	16	Humane endpoint
F	Fecal slurry 1.0 mg/g	72	Experimental endpoint
F	Fecal slurry 1.0 mg/g	16	Humane endpoint
M	Fecal slurry 1.5 mg/g	12	Humane endpoint
M	Fecal slurry 1.5 mg/g	12	Humane endpoint
M	Fecal slurry 1.5 mg/g	12	Humane endpoint
M	Fecal slurry 1.5 mg/g	12	Humane endpoint
M	Fecal slurry 1.5 mg/g	8	Humane endpoint
F	Fecal slurry 1.5 mg/g	12	Humane endpoint
F	Fecal slurry 1.5 mg/g	13	Humane endpoint
F	Fecal slurry 1.5 mg/g	8	Humane endpoint
F	Fecal slurry 1.5 mg/g	12	Humane endpoint
M	Fecal slurry 2.5 mg/g	12	Humane endpoint
M	Fecal slurry 2.5 mg/g	12	Humane endpoint
M	Fecal slurry 2.5 mg/g	12	Humane endpoint
F	Fecal slurry 2.5 mg/g	12	Humane endpoint
F	Fecal slurry 2.5 mg/g	12	Humane endpoint
F	Fecal slurry 2.5 mg/g	16	Humane endpoint
M	Sham	72	Experimental endpoint
M	Sham	72	Experimental endpoint
M	Sham	72	Experimental endpoint
M	Sham	72	Experimental endpoint

F	Sham	72	Experimental endpoint
F	Sham	72	Experimental endpoint
F	Sham	72	Experimental endpoint
F	Sham	72	Experimental endpoint
**Series 2 - Site 1: Fecal Slurry Batch 2021**			
**Sex**	**Treatment**	**Time of death (h)**	
M	Fecal slurry 0.75 mg/g	72	Experimental endpoint
M	Fecal slurry 0.75 mg/g	72	Experimental endpoint
M	Fecal slurry 0.75 mg/g	72	Experimental endpoint
M	Fecal slurry 0.75 mg/g	72	Experimental endpoint
M	Fecal slurry 0.75 mg/g	72	Experimental endpoint
F	Fecal slurry 0.75 mg/g	72	Experimental endpoint
F	Fecal slurry 0.75 mg/g	72	Experimental endpoint
F	Fecal slurry 0.75 mg/g	72	Experimental endpoint
F	Fecal slurry 0.75 mg/g	72	Experimental endpoint
F	Fecal slurry 0.75 mg/g	72	Experimental endpoint
M	Sham	72	Experimental endpoint
M	Sham	72	Experimental endpoint
F	Sham	72	Experimental endpoint
F	Sham	72	Experimental endpoint
**Series 3 - Site 1: Fecal Slurry Batch 2021**			
**Sex**	**Treatment**	**Time of death (h)**	
M	Fecal slurry 0.75 mg/g	36	Died during handling
M	Fecal slurry 0.75 mg/g	48	Found dead
M	Fecal slurry 0.75 mg/g	72	Experimental endpoint
M	Fecal slurry 0.75 mg/g	72	Experimental endpoint
M	Fecal slurry 0.75 mg/g	28	Died during handling
F	Fecal slurry 0.75 mg/g	28	Died during handling
F	Fecal slurry 0.75 mg/g	72	Experimental endpoint
F	Fecal slurry 0.75 mg/g	24	Found dead
F	Fecal slurry 0.75 mg/g	24	Humane endpoint
F	Fecal slurry 0.75 mg/g	72	Experimental endpoint
M	Fecal slurry 0.75 mg/g	24	Humane endpoint
M	Fecal slurry 0.75 mg/g	72	Experimental endpoint
M	Fecal slurry 0.75 mg/g	36	Died during handling
M	Fecal slurry 0.75 mg/g	20	Found dead
M	Fecal slurry 0.75 mg/g	48	Found dead
F	Fecal slurry 0.75 mg/g	72	Experimental endpoint
F	Fecal slurry 0.75 mg/g	72	Experimental endpoint
F	Fecal slurry 0.75 mg/g	16	Found dead
F	Fecal slurry 0.75 mg/g	72	Experimental endpoint
F	Fecal slurry 0.75 mg/g	16	Found dead
M	Sham	72	Experimental endpoint

M	Sham	72	Experimental endpoint
F	Sham	72	Experimental endpoint
F	Sham	72	Experimental endpoint
**Series #3 - Site 2: Fecal Slurry Batch 2021**			
**Sex**	**Treatment**	**Time of death (h)**	
M	Fecal slurry 0.5 mg/g	72	Experimental endpoint
M	Fecal slurry 0.5 mg/g	72	Experimental endpoint
M	Fecal slurry 0.5 mg/g	72	Experimental endpoint
M	Fecal slurry 0.5 mg/g	72	Experimental endpoint
F	Fecal slurry 0.5 mg/g	16	Humane endpoint
F	Fecal slurry 0.5 mg/g	72	Experimental endpoint
F	Fecal slurry 0.5 mg/g	72	Experimental endpoint
F	Fecal slurry 0.5 mg/g	72	Experimental endpoint
M	Fecal slurry 0.625 mg/g	16	Humane endpoint
M	Fecal slurry 0.625 mg/g	16	Found dead
M	Fecal slurry 0.625 mg/g	32	Humane endpoint
M	Fecal slurry 0.625 mg/g	72	Experimental endpoint
F	Fecal slurry 0.625 mg/g	20	Found dead
F	Fecal slurry 0.625 mg/g	20	Humane endpoint
F	Fecal slurry 0.625 mg/g	48	Died during handling
F	Fecal slurry 0.625 mg/g	72	Experimental endpoint
M	Fecal slurry 0.75 mg/g	16	Found dead
M	Fecal slurry 0.75 mg/g	16	Found dead
M	Fecal slurry 0.75 mg/g	16	Found dead
M	Fecal slurry 0.75 mg/g	16	Found dead
F	Fecal slurry 0.75 mg/g	16	Humane endpoint
F	Fecal slurry 0.75 mg/g	24	Humane endpoint
F	Fecal slurry 0.75 mg/g	24	Found dead
F	Fecal slurry 0.75 mg/g	72	Experimental endpoint
M	Sham	72	Experimental endpoint
M	Sham	72	Experimental endpoint
M	Sham	72	Experimental endpoint
F	Sham	72	Experimental endpoint
F	Sham	72	Experimental endpoint
F	Sham	72	Experimental endpoint
**Series #4 - Site 2: Fecal Slurry Batch 2020**			
**Sex**	**Treatment**	**Time of death (h)**	
M	Fecal slurry 0.75 mg/g	72	Experimental endpoint
M	Fecal slurry 0.75 mg/g	72	Experimental endpoint
M	Fecal slurry 0.75 mg/g	72	Experimental endpoint
M	Fecal slurry 0.75 mg/g	72	Experimental endpoint
F	Fecal slurry 0.75 mg/g	72	Experimental endpoint

F	Fecal slurry 0.75 mg/g	72	Experimental endpoint
F	Fecal slurry 0.75 mg/g	72	Experimental endpoint
F	Fecal slurry 0.75 mg/g	72	Experimental endpoint
M	Sham	72	Experimental endpoint
F	Sham	72	Experimental endpoint
